# Early Clinical Predictors of Neurological Outcome in Children With Asphyxial Out-of-Hospital Cardiac Arrest Treated With Therapeutic Hypothermia

**DOI:** 10.3389/fped.2019.00534

**Published:** 2020-01-17

**Authors:** Jainn-Jim Lin, Ying-Jui Lin, Shao-Hsuan Hsia, Hsuan-Chang Kuo, Huei-Shyong Wang, Mei-Hsin Hsu, Ming-Chou Chiang, Chia-Ying Lin, Kuang-Lin Lin

**Affiliations:** ^1^Division of Pediatric Critical Care and Pediatric Neurocritical Care Center, Chang Gung Children's Hospital and Chang Gung Memorial Hospital, Chang Gung University College of Medicine, Taoyuan, Taiwan; ^2^Graduate Institute of Clinical Medical Sciences, College of Medicine, Chang Gung University, Taoyuan, Taiwan; ^3^Division of Pediatric Neurology, Chang Gung Children's Hospital and Chang Gung Memorial Hospital, Chang Gung University College of Medicine, Taoyuan, Taiwan; ^4^Department of Respiratory Therapy, Chang Gung Children's Hospital and Chang Gung Memorial Hospital, Chang Gung University College of Medicine, Taoyuan, Taiwan; ^5^Division of Cardiology, Department of Pediatrics, Kaohsiung Chang Gung Memorial Hospital and Chang Gung University College of Medicine, Kaohsiung, Taiwan; ^6^Division of Critical Care, Department of Pediatrics, Kaohsiung Chang Gung Memorial Hospital and Chang Gung University College of Medicine, Kaohsiung, Taiwan; ^7^Division of Neurology, Department of Pediatrics, Kaohsiung Chang Gung Memorial Hospital and Chang Gung University College of Medicine, Kaohsiung, Taiwan; ^8^Division of Neonatology, Chang Gung Children's Hospital and Chang Gung Memorial Hospital, Chang Gung University College of Medicine, Taoyuan, Taiwan; ^9^Study Group for Intensive and Integrated Care of Pediatric Central Nervous System, Chang Gung Children's Hospital, Taoyuan, Taiwan

**Keywords:** early clinical predictor, asphyxial, pediatric, out-of-hospital cardiac arrest, therapeutic hypothermia

## Abstract

**Aim:** The aim of the current study was to identify early clinical predictors of neurologic outcome in children with asphyxial out-of-hospital cardiac arrest (OHCA) treated with therapeutic hypothermia.

**Methods:** The present retrospective cohort study of comatose children treated with therapeutic hypothermia or normothermia after asphyxial OHCA was conducted between January 2010 and June 2018. All children aged between 1 month and 18 years of age, with a history of at least 3 min of chest compressions were eligible for inclusion. Their 6-month neurological outcomes were evaluated using the Pediatric Cerebral Performance Category (PCPC) score and early clinical predictors were determined.

**Results:** A total of 100 patients met the eligibility criteria for the study. Sixty-four (64%) of the children were male, and the mean age of participants was 4.59 ± 5.45 years. Forty (40%) of the children had underlying disorders. The overall 1-month survival rate was 36%. Only 12 (12%) of the patients had favorable outcomes (PCPC ≤ 2). Thirty-four (34%) of the 100 children were receiving therapeutic hypothermia. In the univariate analysis, an initial lactate level of ≤ 80 mg/dL, a Glasgow coma scale (GCS) score of 5–8, a GCS motor score ≥4 and a present pupil reflex before therapeutic hypothermia, were significantly associated with favorable 6-month neurological outcomes. However, after the multivariate logistic analysis, only initial serum lactate level and GCS before therapeutic hypothermia were significantly associated with favorable 6-month neurological outcomes.

**Conclusion:** Initial serum lactate level and GCS before therapeutic hypothermia were significantly associated with 6-month favorable neurological outcomes in pediatric asphyxial OHCA patients who were treated with therapeutic hypothermia. Therefore, these early clinical predictors could be helpful to facilitate future clinical research in children with asphyxial OHCA treated with therapeutic hypothermia.

## Introduction

Despite advances in resuscitation for pediatric out-of-hospital cardiac arrest (OHCA), pediatric asphyxial OHCA is still associated with high mortality and morbidity. However, to date no therapies have been shown to be effective in ameliorating neurological injuries in pediatric asphyxial OHCA. Therapeutic hypothermia (also called targeted temperature management) improves neurological outcomes in perinatal asphyxia and adult patients with ventricular fibrillation or pulseless ventricular tachycardia. For infants and children who remain comatose after OHCA, advanced pediatric life support guidelines recommend to either maintain 5 days of continuous normothermia (36–37.5°C), or to maintain 2 days of initial continuous hypothermia (32–34°C) followed by 3 days of continuous normothermia (1, 2). However, in a recent publication by the authors, 3 days of therapeutic hypothermia was associated with a better 1-month survival rate and 6-month neurological outcomes compared with 5 days normothermia in pediatric asphyxial OHCA (3).

Early clinical predictors in children with asphyxia OHCA are important for counseling families and making management decisions. The clinical neurological signs (brainstem reflexes and motor response), biochemical markers (e.g., neuronal specific enolase and S100 protein), electrophysiologic tests (somatosensory evoked potentials), and brain imaging (computed tomography or magnetic resonance imaging) have been described as prognostic factors for neurological outcomes (4–6). However, most were identified in adults and are based on studies that preceded the use of therapeutic hypothermia. There have only been several studies conducted after the introduction of therapeutic hypothermia (7–10). In addition, there is limited data on clinical parameters that can be reliably predicted in the first few hours following the onset of asphyxial OHCA (11). Therefore, the aim of the present study was to identify early clinical predictors of neurologic outcome in children with asphyxial OHCA treated with therapeutic hypothermia.

## Materials and Methods

### Patient Population

**Figure 1:**
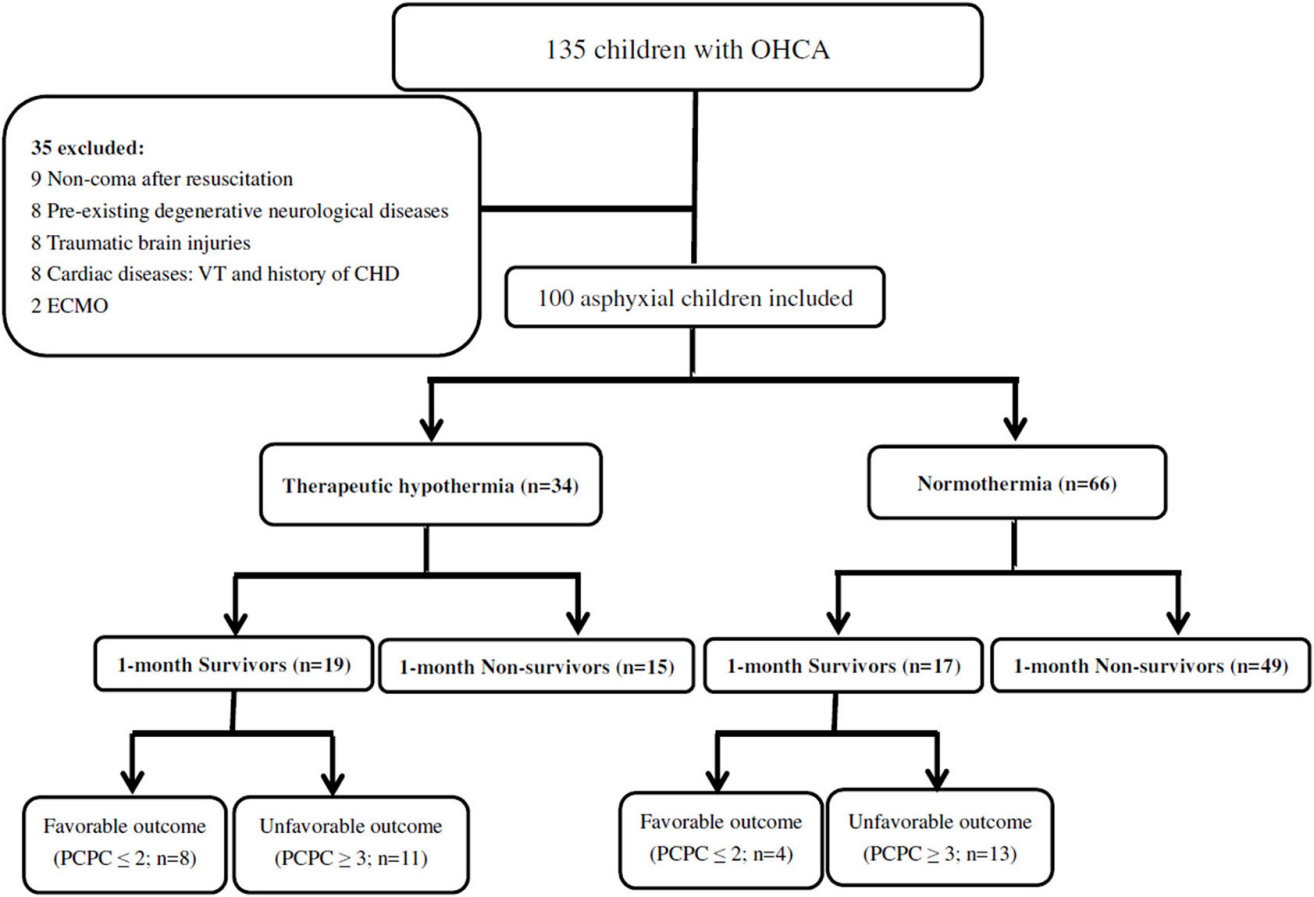
A total of 135 patients with OHCA were identified. 100 asphyxial OHCA comatose patients were enrolled, including 36 patients who survival more than 1 month and 64 patients who died within 1 month. The overall 1-month survival rate was 36%. The patients with 6-month neurological outcomes included those who died during the follow-up period. Eight (23.5%) of the thirty-four patients had favorable outcome (PCPC ≤ 2) in therapeutic hypothermia group and 4 (6.1%) of the 66 patients had favorable outcome (PCPC ≤ 2) in normothermia group. (OHCA, out-of-hospital cardiac arrest; PCPC, pediatric cerebral performance category; VT, Ventricular tachycardia; CHD, congenital heart disease; ECMO, Extracorporeal Membrane Oxygenation).

The current study was a retrospective cohort study that used chart reviews of patients who had been successfully resuscitated, with recovery of spontaneous circulation (ROSC) following asphyxial cardiac arrest. The patients were recruited from two pediatric intensive care units (ICU) at Chang Gung Children's Hospital (Linkou and Kaohsiung branches), between January 1st 2010 and June 30th 2018 (Figure 1). OHCA was defined as patients who received chest compressions before arriving at the hospital (3, 12, 13). Asphyxial cardiac arrest was defined as resuscitation secondary to acute respiratory failure after the evaluation of all available data (3). The criteria for inclusion in the current study were: (1) aged between 1 month and 18 years; (2) duration of cardiac arrest at least 3 min and ROSC after resuscitation; (3) comatose status [Glasgow coma scale (GCS) ≤ 8] after ROSC; and (4) received therapeutic hypothermia or normothermia (3, 12, 13). Patients were excluded if they met any of the following criteria: (1) age > 18 years; (2) not in a coma after resuscitation (GCS > 8); (3) known to have a pre-existing degenerative neurological disease; (4) with a traumatic brain injury; (5) cardiogenic etiologies (ventricular fibrillation or a history of congenital heart disease); and (6) on extracorporeal membrane oxygenation. In therapeutic hypothermia group, all patients received therapeutic hypothermia within 6 h of their resuscitation (3, 12). Because this was a retrospective study, the need for informed consent was waived from all participants in the study. The present study was approved by the Chang Gung Memorial Hospital Institutional Review Board (IRB numbers: 201700975B0, 201700976B0, 201700977B0, and 201900302B0).

### Focused Neurological Examination and Therapeutic Hypothermia Protocol

Patients were initially treated in the emergency department and then transferred to the pediatric ICU after stabilization. Focused neurological assessments, including GCS, pupillary light responses and motor responses to noxious stimulation were performed by physicians in the emergency department after ROSC and by pediatric ICU physicians before the start of therapeutic hypothermia. Pupil responses were scored as present if they were detected in either eye. Motor responses were scored as present if they were detected in any extremity with any degree of stimulation (*M* ≥ 4) (5, 14).

The cooling methods and treatment protocol for therapeutic hypothermia in the present study have been published previously (3, 12, 13). In brief, therapeutic hypothermia to 33°C was induced using thermal heat exchange cooling pads (Arctic Sun, Medivance Inc. Louisville, CO, USA) and maintained for 72 h. Patients were then slowly rewarmed by 1°C per day until the patient reached 36°C.

### Data Collection

The following information was collected from all patients: (1) demographics and pre-existing diseases; (2) event characteristics during cardiopulmonary resuscitation; (3) variables after resuscitation, such as initial serum lactate level, post-cardiac arrest GCS and focused neurological examinations; and (4) outcomes. Initial blood samples were collected within 1 h of ROSC. The primary outcome was neurological outcome, which was assessed using Pediatric Cerebral Performance Category (PCPC) scores in children who survived for 6 months after the events and in those who died during follow-up (3, 12, 13). PCPC scores measure the degree of cognitive function and are recorded as 1–6, where 1 is normal, 2 indicates mild disability, 3 indicates moderate disability, 4 indicates severe disability, 5 indicates coma or vegetative state, and 6 indicates brain death. Neurological outcomes were dichotomized as either a favorable prognosis (PCPC ≤ 2) or unfavorable prognosis (PCPC ≥ 3). The secondary outcomes were duration of hospitalization and the survival rate at 30 days.

### Statistical Analysis

The patients were divided into therapeutic hypothermia or normothermia group. Patient characteristics are represented as descriptive statistics, and the data are presented as mean ± standard deviation. Between group differences were analyzed using the chi-squared test or Fisher's exact test for categorical variables, and Student's *t*-tests for normally distributed continuous variables. The Mann-Whitney *U*-test was used for non-normally distributed data.

Associations between the clinical parameters and 6-month favorable neurological outcomes were determined using univariate analyses. All variables associated with favorable neurological outcomes in the univariate analysis were included in a multiple logistic regression model as candidate predictors. The receiver operating characteristic (ROC) curve for initial serum lactate level was plotted to predict the 6-month neurological outcome. The respective areas under the ROC curves and cut-off points were calculated. Statistical analysis was performed using SPSS statistical software, version 23.0 (IBM, Inc., Chicago, IL, USA). A two-sided *p-*value of <0.05 was considered to indicate a statistically significant difference.

## Results

### Patient Profile and Outcome

A total of 135 OHCA patients were identified during the 7.5-year study period, of whom 100 (74.1%) met the inclusion criteria. A total of 35 children were excluded, including nine with non-coma after resuscitation, eight with pre-existing degenerative neurological diseases, eight with traumatic brain injuries, six with ventricular tachycardia, two with a history of congenital heart disease, and two who received extracorporeal membrane oxygenation (Figure 1). Thirty-four were receiving therapeutic hypothermia and 66 were receiving normothermia therapy.

Of the 100 included children, 64 (64%) were male, and 64 (64%) were <4 years old; the mean age of participants was 4.59 ± 5.45 years. Forty (40%) of the children had underlying disorders. Sixty-three (63%) of the events were bystander-witnessed cardiac arrests, however only 35 (55.6%) of the 63 bystanders performed cardiopulmonary resuscitation (CPR). The first documented arrest rhythm was described as asystole in 92 (92%) patients and bradycardia/pulseless electrical activity in 8 (8%) patients. There were no significant differences in the demographic data, including age, gender, the presence of chronic illnesses, bystander-witnessed cardiac arrest, bystander performed CPR, and first documented arrest rhythm, between the favorable and unfavorable outcome groups in the therapeutic hypothermia and normothermia. The demographic data and 6-month neurological outcomes are listed in Table 1.

**Table 1:** Characteristics of the 100 children with asphyxial out-of-hospital cardiac arrest receiving therapeutic hypothermia or normothermia.

	**Therapeutic hypothermia (*****n*** **=** **34)**			**Normothermia (*****N*** **=** **66)**		
**Characteristic**	**Favorable outcome (*n* = 8)**	**Unfavorable outcome (*n* = 26)**	***p*-value**	**Favorable outcome (*n* = 4)**	**Unfavorable outcome (*n* = 62)**	***p*-value**
Gender			1.000			0.413
Female	2 (25%)	5 (19.2%)		1 (25%)	28 (45.2%)	
Male	6 (75%)	21 (80.8%)		3 (75%)	34 (54.8%)	
Age			0.715			0.311
1–11 months	5 (62.5%)	13 (50%)		1 (25%)	26 (41.9%)	
1–4 years	2 (25%)	5 (19.2%)		0 (0%)	12 (19.4%)	
5–8 years	0 (0%)	3 (11.5%)		0 (0%)	5 (8.1%)	
9–18 years	1 (12.5%)	5 (19.2%)		3 (75%)	19 (30.6%)	
Chronic pre-existing illness			0.769			0.039
No	6 (75%)	16 (61.5%)		1 (25%)	37 (59.7%)	
Respiratory	0 (0%)	3 (11.5%)		0 (0%)	11 (17.7%)	
Neurologic	1 (12.5%)	3 (11.5%)		3 (75%)	10 (16.1%)	
Other	1 (12.5%)	4 (15.5%)		0 (0%)	4 (6.5%)	
Bystander-witnessed cardiac arrest	6 (75%)	13 (50%)	0.257	3 (75%)	41 (66.1%)	1.000
Bystander performed CPR	3 (37.5%)	8 (30.8%)	1.000	1 (25%)	23 (37.1%)	1.000
Initial rhythm			0.131			0.555
Asystole	6 (75%)	25 (100%)		4 (100%)	57 (91.9%)	
Bradycardia/PEA	2 (25%)	1 (0%)		0 (0%)	5(8.1%)	

*CPR, cardiopulmonary resuscitation; PEA, pulseless electrical activity*.

*Favorable outcome: pediatric cerebral performance category ≤ 2; Unfavorable outcome: pediatric cerebral performance category ≥3*.

The overall 1-month survival rate was 36%. However, only 12 (12%) of the 100 patients had favorable outcomes (PCPC ≤ 2). The mean duration of ICU and hospitalization were 24.24 ± 37.61 and 25.42 ± 37.98 days, respectively.

### Association of Variables During and After Resuscitation and Patient Outcomes in Therapeutic Hypothermia

**Figure 2:**
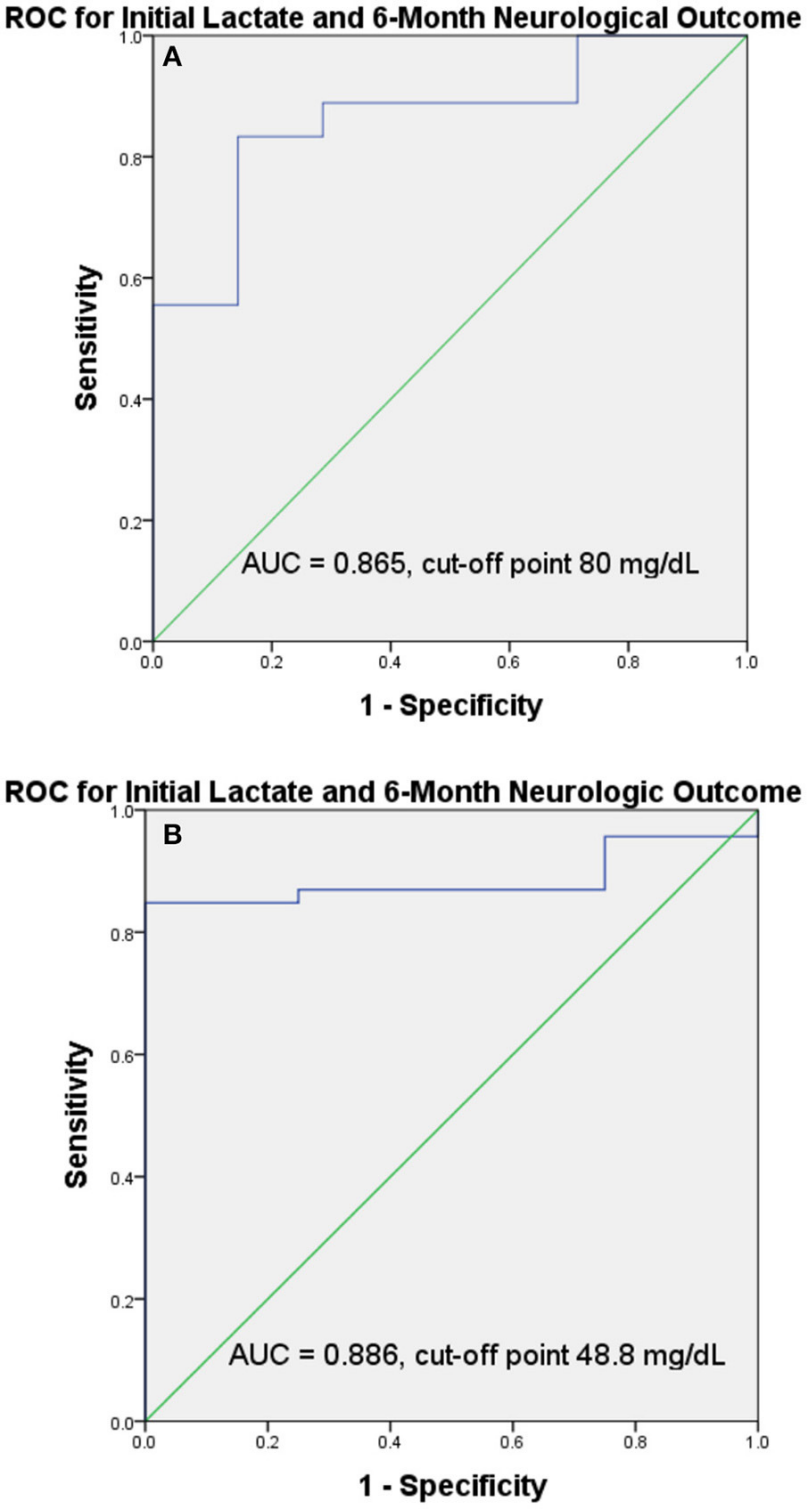
ROC curves and the AUC of the ROC curves and cut-off points for initial serum lactate level. The AUC of the ROC and cut-off points showed that initial serum lactate level had good discriminative power in predicting favorable 6-month neurological outcomes in therapeutic hypothermia group (AUC = 0.865, cut-off point 80 mg/dL) **(A)** and in normothermia group (AUC = 0.886, cut-off point 48.8 mg/dL) **(B)**. (AUC, area under the curve; ROC, receiver operating characteristic).

The mean duration of cardiac arrest until the ROSC was 23.56 min (range 8–81 min). After resuscitation, initial serum lactate levels were significantly higher in the unfavorable outcome group (119.35 ± 53.85 mg/dL) compared with the favorable outcome group (59.72 ± 27.61 mg/dL; *p* = 0.011). Areas under the ROC curves for initial serum lactate levels after resuscitation for predicting the favorable 6-month neurological outcomes, are shown in Figure 2A. The areas under the ROC curves for initial serum lactate levels after resuscitation in predicting favorable outcome was 0.865 and the cut-off point was 80 mg/dL. The duration of cardiac arrest until the ROSC, as well as the serum pH, glucose, and creatinine levels immediately after resuscitation, were similar between the two outcome groups. The event characteristics during resuscitation and 6-month neurological outcomes are listed in Table 2.

**Table 2:** Variables during and after resuscitation of the 100 asphyxial out-of-hospital cardiac arrest children receiving therapeutic hypothermia and normothermia.

	**Therapeutic hypothermia (*****n*** **=** **34)**			**Normothermia (*****n*** **=** **66)**		
**Characteristics**	**Favorable outcome (*n* = 8)**	**Unfavorable outcome (*n* = 26)**	***p*-value**	**Favorable outcome (*n* = 4)**	**Unfavorable outcome (*n* = 62)**	***p*-value**
**Characteristics during resuscitation**						
Interval of CPR to ROSC (min)	18.75 ± 9.54	25.04 ± 15.42	0.286	5.50 ± 0.70	32.28 ± 22.08	0.096
Seizure	4 (50%)	11 (42.3%)	1.000	2 (50%)	15 (24.2%)	0.271
Serum pH	7.226 ± 0.135	7.039 ± 0.261	0.063	7.220 ± 0.528	7.052 ± 0.281	0.001^*^
Initial glucose (mg/dL)	203.38 ± 95.50	266.74 ± 140.56	0.249	156.75 ± 84.59	266.81 ± 161.32	0.185
Initial lactate (mg/dL)	59.72 ± 27.61	119.35 ± 53.85	0.011^*^	29.00 ± 14.89	113.56 ± 67.54	<0.001^*^
Admission creatinine (mg/dL)	0.53 ± 0.50	1.16 ± 1.30	0.066	0.68 ± 0.34	0.84 ± 0.67	0.649
**Focused neurologic examination**						
**Initial examination after ROSC**						
GCS	3.71 ± 1.23	3.52 ± 1.29	0.726	3.50 ± 1.00	3.26 ± 0.80	0.569
GCS motor score ≥4	2 (25%)	3 (11.5%)	0.570	0(0%)	2 (3.2%)	1.000
Present pupil reflex	4 (50%)	6 (23.1%)	0.195	2 (50%)	8 (12.9%)	0.106
**Examination before therapeutic hypothermia or 4 h after admission in normothermia**						
GCS	5.75 ± 1.90	3.65 ± 1.29	0.017^*^	4.50 ± 1.91	3.22 ± 1.16	0.047^*^
GCS≥5	6 (75%)	4 (15.4%)	0.003^*^	2 (50%)	2 (3.2%)	0.016^*^
GCS motor score ≥4	5 (62.5%)	4 (15.4%)	0.017^*^	1 (25%)	2 (3.2%)	0.174
Present pupil reflex	6 (75%)	7 (26.9%)	0.033^*^	4 (100%)	9 (14.5%)	0.001^*^
**Outcomes**						
ICU stay	31.88 ± 45.55	29.62 ± 41.14	0.895	55.75 ± 37.25	18.97 ± 34.38	0.043^*^
Hospital length of stay (days)	38.50 ± 45.51	31.42 ± 41.40	0.682	59.50 ± 36.30	19.02 ± 34.36	0.026^*^

*CPR, cardiopulmonary resuscitation; ROSC, return of spontaneous circulation; GCS, Glasgow Coma Scale; PCPC, pediatric cerebral performance category*.

*Favorable outcome: PCPC ≤ 2; Unfavorable outcome: PCPC ≥ 3*.

* *p < 0.05: statistically significant*.

### Association Between Focused Neurological Examinations and Patient Outcomes in Therapeutic Hypothermia

In terms of the initial examination after the ROSC, no findings were significantly associated with 6-month favorable neurological outcomes. However, in terms of the examination before therapeutic hypothermia, a GCS score 5–8, a GCS motor score ≥4 and the presence of a pupil reflex, were all significantly associated with 6-month favorable neurological outcomes (*p* = 0.003, 0.017, and 0.033, respectively). The focused neurological examinations after resuscitation and before therapeutic hypothermia, and the 6-month neurological outcomes are listed in Table 2.

### Multivariate Analysis of Clinical Parameters on Therapeutic Hypothermia Group for 6-Month Neurological Outcomes

The univariate and multivariate analysis of clinical parameters and 6-month favorable neurological outcomes are shown in Table 3. The univariate analysis revealed four clinical parameters were significantly associated with 6-month favorable neurological outcomes, including the initial serum lactate level, the GCS, a GCS motor score ≥4 and the presence of a pupil reflex before therapeutic hypothermia. As there is a strong correlation between the GCS and the GCS motor score, a two model multivariable logistic regression was used. In model 1, initial serum lactate levels and the GCS before therapeutic hypothermia were entered in the logistic regression analysis. In the multivariate logistic regression model, initial serum lactate levels and GCS were significantly associated with 6-month favorable neurological outcomes. In model 2, the initial serum lactate level, q GCS motor score ≥4 and the presence of a pupil reflex before therapeutic hypothermia were entered into the logistic regression analysis. However, in the multivariate logistic regression model, not all characteristics were significantly associated with a 6-month favorable neurological outcome.

**Table 3:** Clinical Predictors for 6-month favorable neurological outcomes in children with asphyxial out-of-hospital cardiac arrest who received therapeutic hypothermia.

**Characteristics**	**Favorable outcome (*n* = 8)**	**Unfavorable outcome (*n* = 26)**	**Univariate analysis**			**Multi-variate analysis**		
			**Odd ratio**	**95% CI**	***p*-value**	**Odd ratio**	**95% CI**	***p*-value**
**Model 1**								
Initial lactate level	59.72 ± 27.61	119.35 ± 53.85	0.954	0.914–0.995	0.030^*^	0.941	0.886–0.999	0.048^*^
GCS^++^	5.75 ± 1.90	3.65 ± 1.29	2.079	1.230–3.515	0.006^*^	2.179	1.058–4.486	0.035^*^
**Model 2**								
Initial lactate level	59.72 ± 27.61	119.35 ± 53.85	0.954	0.914–0.995	0.030^*^	0.933	0.869–1.002	0.056
GCS motor score ≥4^++^	5 (62.5%)	4 (15.4%)	9.16	1.539–54.592	0.015^*^	33.689	0.782–1451.585	0.067
Present pupil reflex^++^	6 (75%)	7 (26.9%)	8.14	1.320–50.250	0.024^*^	1.007	0.044–20.051	0.966

*GCS, Glasgow coma scale; CI, confidence interval*.

*Favorable outcome: pediatric cerebral performance category ≤ 2; Unfavorable outcome: pediatric cerebral performance category ≥3*.

++ *Examination before therapeutic hypothermia*.

* *p < 0.05: statistical significant*.

### Association of Clinical Parameters on Normothermia Group for 6-Month Neurological Outcomes

A total of 66 patients received normothermia therapy. The overall 1-month survival rate was 25.7%. Only 4 (6.1%) of the 66 patients had favorable outcomes (PCPC ≤ 2). After resuscitation, initial serum lactate levels were significantly higher in the unfavorable outcome group (113.56 ± 67.54 mg/dL) compared with the favorable outcome group (29.00 ± 14.89 mg/dL; *p* < 0.001). The areas under the ROC curves for initial serum lactate levels after resuscitation in predicting favorable outcome was 0.886 and the cut-off point was 48.8 mg/dL (Figure 2B). In the univariate analysis, the initial lactate level of ≤ 48.8 mg/dL, a GCS score of 5–8, and a present pupil reflex 4 h after admission were significantly associated with favorable 6-month neurological outcomes. However, after the multivariate logistic analysis, no of them were significantly associated with favorable 6-month neurological outcomes.

## Discussion

It is important to identify pediatric asphyxial OHCA patients who can benefit from therapeutic hypothermia. However, at present there is limited data on the clinical parameters that can be reliably predicted in the first few hours after asphyxia OHCA, in children that might benefit from therapeutic hypothermia (11). One of the most widely accepted indicators is a GCS score ≤ 8. THAPCA Trial, the largest trials of target temperature management in children after cardiac arrest, excluded patient with a score of 5 or 6 on the GCS motor-response subscale and their result showed in comatose children who survived OHCA, therapeutic hypothermia, as compared with therapeutic normothermia, did not confer a significant benefit in survival with a good functional outcome at 1 year (2). In the current study, an initial lactate level ≤ 80 mg/dL and a GCS score 5–8 before therapeutic hypothermia, were associated with a 6-month favorable neurological outcome in pediatric asphyxial OHCA, which was treated with therapeutic hypothermia. However, in normothermia group, the initial lactate level of ≤ 48.8 mg/dL was associated with a 6-month favorable neurological outcome. These initial clinical parameters demonstrate the same relationship in the normothermia and therapeutic hypothermia group and could be related to arrest severity and hence predict outcome. Further prospective studies are needed to confirm this finding.

The prediction of pre-hospital variables in children with asphyxial OHCA who may benefit from therapeutic hypothermia is unknown. Okada et al. developed the 5-R scoring system (initial rhythm with ventricular fibrillation or ventricular tachycardia, starting resuscitation ≤ 5 min, ROSC ≤ 30 min, recovery of pupillary light reflex, absence of re-arrest), and demonstrated that the 5-R score was associated with a good neurological outcome in patients treated with therapeutic hypothermia in adult OHCA (7). Choi et al. conducted a retrospective observational study using a national OHCA cohort database, and they concluded that the effect of therapeutic hypothermia was greatest in witnessed OHCA patients with pulseless electrical activity as the initial rhythm (15). Goto et al. conducted a prospective observational study using the Japanese national pediatric OHCA cohort database and developed the decision tree model using three pre-hospital variables: pre-hospital ROSC, initial shockable rhythm and witnessed arrest (11). However, in the present study, there were no significant differences in pre-hospital variables, including bystander-witnessed cardiac arrest, bystander performed CPR and first documented arrest rhythm, between the favorable and unfavorable outcome groups. Therefore, the reliability of pre-hospital variables in children with asphyxial OHCA who may benefit from therapeutic hypothermia remains unknown.

Serum lactate level is a good prognostic indicator for tissue perfusion deficit. It has recently been reported that blood lactate level measured on arrival is useful as a prognostic predictor for cardiac arrest in adult and neonates who undergo therapeutic hypothermia (16–19). Lee et al. reported that high serum lactate levels measured within 1 h of ROSC, were positively associated with hospital mortality and poor neurological outcomes in 443 adult OHCA patients treated with therapeutic hypothermia (17). A systematic review and meta-analysis also showed that high serum lactate levels on admission were associated with poor neurological outcomes after cardiac arrest, whereas the association between serum lactate clearance and neurological outcomes was not so stable (18). In the present study, it was found that an initial lactate level ≤ 80 mg/dL within 1 h of ROSC was positively associated with a 6-month favorable neurological outcome in pediatric asphyxial OHCA treated with therapeutic hypothermia. Therefore, initial serum lactate level ≤ 80 mg/dL appears to be a good biomarker for predicting children who may benefit from therapeutic hypothermia in a clinical setting.

The prognostic value of neurological examinations after cardiac arrest have been previously described, however most were based on studies that preceded the use of therapeutic hypothermia (4–6). The American Academy of Neurology practice parameters concluded that absent pupillary, corneal and motor responses 3 days after adult cardiac arrest were predictive of poor neurological outcomes (20). However, these guidelines did not address the pediatric age group. Only a prospective cohort study of children treated with therapeutic hypothermia after cardiac arrest, including cardiac and asphyxial etiologies, demonstrated that absent motor and pupil responses soon after ROSC were not predictive of unfavorable outcomes (14). The current study focused on asphyxial OHCA in children. During the univariate analysis, GCS score 5–8, GCS motor score ≥4 and the presence of a pupil reflex before therapeutic hypothermia were significantly associated with a 6-month favorable neurological outcome. However, after multivariate logistic analysis, only GCS before therapeutic hypothermia was associated with favorable 6-month neurological outcomes.

## Limitations

There were several limitations to the present study. First, it was a retrospective study in a limited cohort of children after resuscitation. There were only 34 patients receiving therapeutic hypothermia over 7.5 years and not all data was available. The number of patients with positive clinical outcomes is only eight in therapeutic hypothermia. This may lead to recording bias and poor quality of the final data. However, very few reports in pediatric literature have discussed early clinical predictors in children receiving therapeutic hypothermia after asphyxial OHCA, and the novelty of this study may outweigh the limitation of the small number of cases. Second, therapeutic hypothermia in the current study was performed in two pediatric intensive care units in the hospital branches. Although the general principles of post-cardiac arrest care were similar, possible confounders, such as the use of vasopressors, strategy of ventilator support, sedation, and anesthesia were not investigated with respect to improving the outcomes of post-cardiac arrest care. Comparing the findings directly with other reports should take this into account. Future studies should address these possible confounders.

## Conclusion

Initial serum lactate level ≤ 80 mg/dL and GCS score 5–8 before therapeutic hypothermia were significantly associated with a 6-month favorable neurological outcome for pediatric asphyxial OHCA treated with therapeutic hypothermia. Therefore, these early clinical predictors could be helpful to facilitate future clinical research in children with asphyxial OHCA treated with therapeutic hypothermia. Further prospective studies are necessary to confirm this finding.

## Data Availability Statement

All datasets generated for this study are included in the article/supplementary material.

## Ethics Statement

The studies involving human participants were reviewed and approved by the Chang Gung Memorial Hospital Institutional Review Board (IRB numbers: 201700975B0, 201700976B0, 201700977B0 and 201900302B0). Written informed consent from the participants' legal guardian/next of kin was not required to participate in this study in accordance with the national legislation and the institutional requirements.

## Members of the Consortium/Group

The authors' names and affiliations of the iCNS Study Group are as follows: Kuang-Lin Lin (lead author, Division of Pediatric Neurology, Chang Gung Children's Hospital, Linkou branch; lincgh@cgmh.org.tw); Huei-Shyong Wang, I-Jun Chou, Yi-Shan Wang (Division of Pediatric Neurology, Chang Gung Children's Hospital, Linkou branch); Jainn-Jim Lin, Shao-Hsuan Hsia, Chia-Ying Lin (Division of Pediatric Critical Care Medicine, Chang Gung Children's Hospital, Linkou branch); Ying-Jui Lin, Hsuan-Chang Kuo, Mei-Hsin Hsu (Division of Pediatric Critical Care Medicine, Chang Gung Children's Hospital, Kaohsiung branch); Ming-Chou Chiang, Reyin Lien (Division of Neonatalogy, Chang Gung Children's Hospital, Linkou branch); Ai-Hua Yeh, Wei-Chun, Lin, Fang-Yu Hsu, Yi-Ling Huang (Clinical Research Team of Pediatric Critical Care Medicine, Chang Gung Children's Hospital, Linkou branch).

## Author Contributions

J-JL conceived the study. J-JL, Y-JL, S-HH, H-CK, M-HH, M-CC, and C-YL participated in data collection. K-LL and H-SW participated in the study's design and coordination. J-JL and Y-JL drafted the manuscript. K-LL critically revised the manuscript for important intellectual content.

### Conflict of Interest

The authors declare that the research was conducted in the absence of any commercial or financial relationships that could be construed as a potential conflict of interest.
